# Using mass-media communications to increase population usage of Australia’s Get Healthy Information and Coaching Service®

**DOI:** 10.1186/1471-2458-12-762

**Published:** 2012-09-11

**Authors:** Blythe J O’Hara, Adrian E Bauman, Philayrath Phongsavan

**Affiliations:** 1 Prevention Research Collaboration, Sydney School of Public Health, University of Sydney, Medical Foundation Building K25, Sydney, NSW 2006, Australia

**Keywords:** Mass-media, Recruitment, Telephone-based counselling, Lifestyle intervention

## Abstract

**Background:**

Global obesity prevalence is increasing and population health programs are required to support changes to modifiable lifestyle risk factors. Such interventions benefit from mass-communications to promote their use. The Get Healthy Information and Coaching Service ® (GHS) utilised mass-reach media advertising to recruit participants to an Australian state-wide program.

**Methods:**

A stand alone population survey collected awareness, knowledge and behavioural variables before the first advertising phase, (n = 1,544; August -September 2010), during (n = 1,500; February - March 2011) and after the advertising period (n = 1,500; June-July 2011). GHS usage data (n = 6,375) was collated during July 2010 – June 2011.

**Results:**

The results showed that television-lead mass-media significantly increased unprompted awareness (0% to 31.8%, p < 0.001); prompted awareness (2.5% to 23.7%, p < 0.001); and understanding (10.2% to 32.2%, p < 0.001). Mass-media (television, print and mail out information) was more often cited as the source of referral by males, those aged 18 – 49 years, employed, and from the lowest socio-economic groups. During the weeks when mass-media advertising was present, 4 and 2.5 times more information and coaching participants respectively registered than when there was no advertising present. Participants who cited television and print were less likely to enrol in GHS coaching, but this was not the case for mail out information and secondary referral sources.

**Conclusions:**

GHS mass-communications campaigns are effective at increasing awareness and usage of the GHS, especially among hard-to-reach population groups. Television advertising provides universal reach, but should be supplemented by health professional referrals and targeted mail-out information to recruit participants to the intensive GHS coaching program.

## Background

In Australia nearly two-thirds of the adult population are overweight or obese [1,2], with high associated direct and indirect costs in the order of $21 billion annually [3]. Increased energy intake and reduced energy expenditure are primary contributory factors in obesity increases. Population wide programs are needed to support healthy eating and increased physical activity.

Systematic reviews have confirmed that telephone-based interventions [4,5] are effective in increasing physical activity, improving nutrition and reducing weight. Within this context, in 2009 the state of New South Wales (NSW) launched the Get Healthy Information and Coaching Service® (GHS), a free Government-funded, population-based telephone service aimed at helping adults achieve and maintain lifestyle based changes ( http://www.gethealthynsw.com.au). The GHS targets the 52.2% of NSW adults who are overweight or obese; the 45.4% of NSW adults who do not undertake the recommended levels of physical activity; and the proportion of adults who do not eat the recommended levels of fruit and vegetables (49.6% and 91.3% respectively) [6]. Callers to the GHS can receive detailed information on physical activity, nutrition and healthy weight and/or enrol in the intensive and evidence-based 6-month coaching (counselling) program that provides ongoing support to assist them in setting and achieving their health related goals.

The GHS was launched and promoted using both state-level GHS specific mass-reach campaigns (February 2009 – June 2009) and indirectly through a national healthy weight mass-media campaign ( http://www.measureup.gov.au) that included the GHS phone number as a call to action. From July 2010 to June 2011, the GHS was promoted again using the GHS-specific campaign and using the GHS tag line nested within the next phase of the national healthy weight campaign ( http://www.swapit.gov.au). The GHS-specific campaign included television advertising (two 30-second advertisements and a 15-second advertisement) encouraging people to think about healthier lifestyles and directed people to call the GHS. Supportive marketing activities included radio, print, online advertising and pamphlets being distributed in household letterboxes and home delivered magazines specifically targeted to disadvantaged locations (based on geography and unhealthy lifestyle behaviours). The national healthy weight campaign encouraged the swapping of unhealthy behaviours for healthy ones and prompted viewers to call the GHS phone number.

The use of public education campaigns to catalyse people to think about behaviour change is well understood [7-9]. The effectiveness of mass-media campaigns in increasing health service utilisation is also recognized [10] and can be effective at promoting awareness and use of a new service or program [11-13]. Such a relationship is well established with mass-media being widely used to promote calls to telephone-based smoking cessation services such as Tobacco Quitlines [14-17]. However, the differential effects of mass-media on accessing Quitlines service levels (eg., quitting information via the mail vs. comprehensive, proactive multi-session behaviour modification programs [18]) are largely unknown. With respect to lifestyle-change programs, limited evidence exists on the relative impact of mass-media campaigns on driving people to different program components, although a previous study has reported that continuous advertising of the GHS state-wide prevention initiative achieves good reach and recall among socio-economically disadvantaged groups [19].

The question remains as to the most effective way to increase the population reach of a lifestyle-based telephone counselling service. To answer this question, this paper examines the effectiveness of the mass-reach media campaign on increasing awareness of the GHS and its usage. This paper also examines the associations between the type of media, referral source and GHS usage; in particular what source of referral is most effective at recruiting participants to enrol in the intensive 6-month GHS coaching program.

## Methods

### Get Healthy specific and “Swap it don’t stop it” advertising

**Figure 1:**
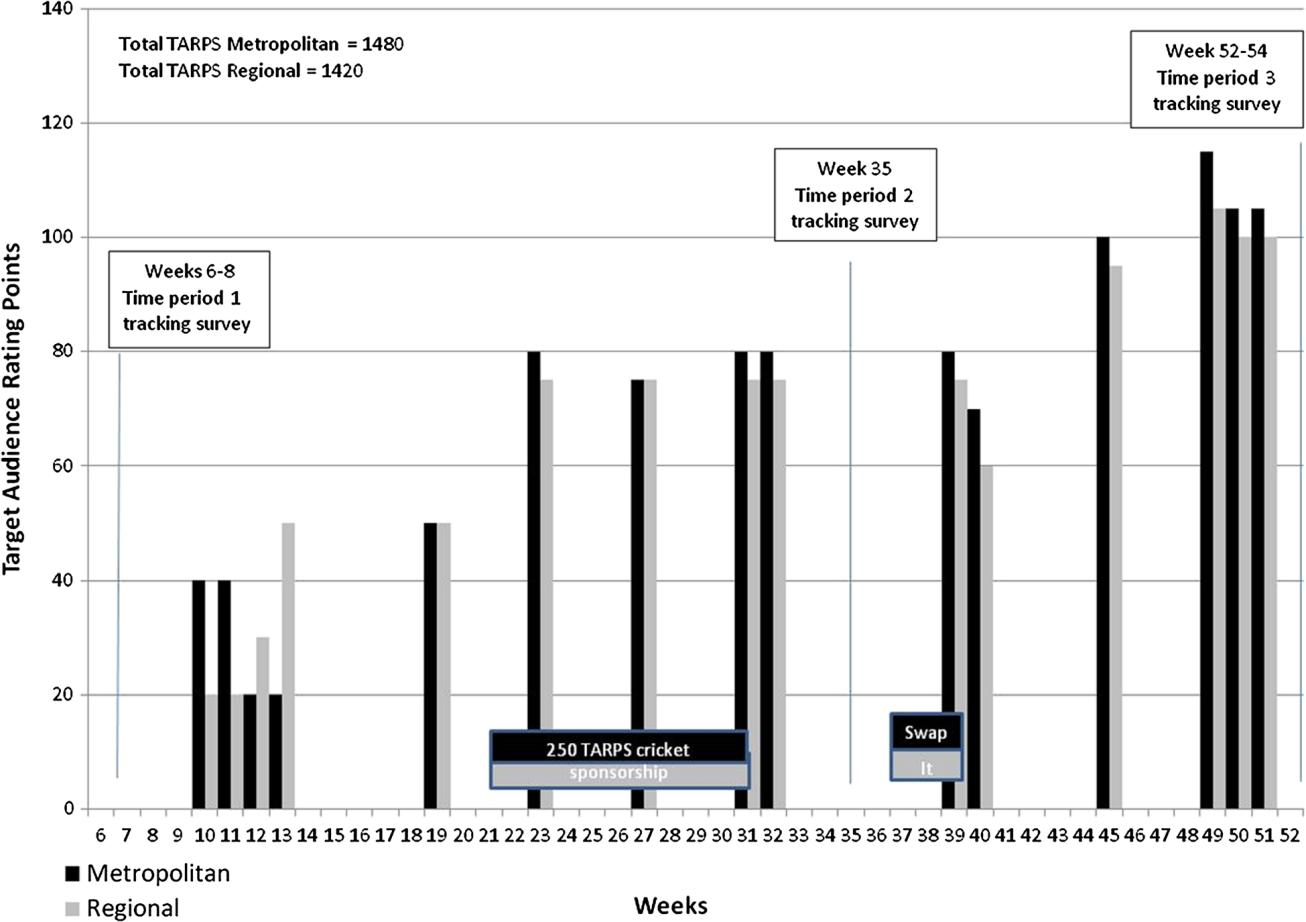
Metropolitan and Regional television advertising for the period August 2010 – June 2011.

The GHS mass-media advertising targeted adults aged 25 – 54 years. The first wave of mass-media advertising to promote the GHS was from September 2010 to June 2011. Campaign activities included: television advertisements (15 and 30 second messages; 60:40 peak:off peak television programming)(Figure 1); metropolitan, suburban and primary regional print advertisements; Culturally and Linguistically Diverse print titles; Indigenous print titles; live radio reads on Australian Traffic Network (metropolitan and major regional locations); online advertising and brochure inserts into an “open road” magazine (a bi-monthly subscription magazine that has articles relevant to motorists, car advice, and travel stories and ideas) delivered to 522,000 households across regional NSW, and; letterbox drops into 309,000 households in disadvantaged neighbourhoods across NSW (Table 1).

**Table 1:** Weeks and type of advertising in July 2010- June 2011

			
***week 1 - 9***	***Time period 1 tracking survey;*** no advertising	***week 31***	TV, press & online
***week 10***	TV, print, radio & online	***week 32***	TV, press & online
***week 11***	TV & online advertising	***week 33***	online
***week 12***	TV, print, radio & online	***week 34***	no advertising
***week 13***	TV & online	***week 35***	***Time period 2 tracking survey;*** no advertising
***week 14***	online	***week 36***	Letter box drops & magazine inserts in prescribed geographical locations
***week 15***	online	***week 37***	Letter box drops & magazine inserts & "Swap it" branding
***week 16***	online	***week 38***	Magazine inserts, press, online & "Swap it" branding
***week 17***	online, print & radio	***week 39***	TV, magazine inserts, press, online & "Swap it" branding
***week 18***	online advertising	***week 40***	TV, magazine inserts, press, online & "Swap it" branding
***week 19***	TV, print, radio & online	***week 41***	Print & online
***week 20***	no advertising	***week 42***	print
***week 21***	TV & print	***week 43***	print
***week 22***	TV	***week 44***	print
***week 23***	TV & print	***week 45***	TV & print & online
***week 24***	TV	***week 46***	Print & online
***week 25***	TV	***week 47***	Print & online
***week 26***	TV	***week 48***	Print & online
***week 27***	TV	***week 49***	TV & print & online
***week 28***	TV and online	***week 50***	TV & print
***week 29***	TV and online	***week 51***	TV & print
***week 30***	TV and online	***week 52***	***Time period 3 tracking survey*;** no advertising
**Total advertising throughout the year**			
***Print advertisements***	· 26 in metropolitan press titles		· 75 in culturally and linguistically diverse press titles
	· 104 in suburban press titles		
	· 205 in primary regional press titles		· 10 in Aboriginal press titles
***Online advertisements***	· 15 sec pre roll advertising; behavioural and demographic targeted advertising; direct traffic and sponsored links		
***Letterbox drops and Magazine inserts***	· 309,000 letter box drops; 271,254 metropolitan households and 37,087 regional households specifically targeted to disadvantaged locations (based on geography and unhealthy lifestyle behaviours)		
	· 522,000 inserts into “open road”, 161,000 in Newcastle and Hunter region, 121,000 in North Coast region, 69,000 Southern Tablelands and Central West NSW and 109,000 South Coast NSW.		
***Radio***	· 440 “live read” 10 sec advertisements in metropolitan Sydney		
	· 200 “live read” 10 sec advertisements in regional locations (Central Coast, Newcastle, Nowra, Lithgow and Wollongong		

In NSW, the promotion of the GHS was at the end of “Swap it don’t stop it” television advertisements which had been aired for four weeks in March 2011. This television advertisement promotes the notion of swapping unhealthy behaviours for healthier choices to decrease waist circumferences, eg. swapping the lift for the stairs.

### Campaign tracking survey

To evaluate the effects of the September 2010 - June 2011 campaign, a state-wide baseline survey was undertaken in August – September 2010 (time period 1), a follow up survey in February – March 2011 (time period 2); and further follow up survey in June – July 2011 (time period 3). The NSW Ministry for Health engaged a market research agency to undertake this standalone population telephone tracking survey. The target population for the advertising tracking survey was English-speaking NSW adults, aged 18 years and older living in private dwellings with telephone landlines. The households were randomly sampled and respondents were recruited based on the “last birthday” selection method using Random Digit Dialling. The sample size was n = 1,500 for each survey.

### Survey measures

Information was collected via telephone-based interviews on socio-demographic variables (age, gender, geographical location, education, household composition, employment status, income, language spoken at home, Indigenous status, and postcode); self-reported health, weight (kg), height (cm); campaign recall (where participants were asked to describe any healthy lifestyle advertising they had seen), campaign recognition (where participants were described the advertising and asked about recognition), message take-out, actions as a result of seeing the advertising and perceived service effectiveness and personal relevance. The survey took less than 10 minutes to complete.

### GHS usage data

To examine which referral pathways were associated with GHS service levels, participant usage data from July 2010 to June 2011 was examined. Participants were classified according to service levels: information-only participants who received (via mail or e-mail) an information package on healthy eating [20], physical activity [21], and achieving or maintaining a healthy weight; coaching participants who received 10 individually-tailored calls to support behaviour change [22-24], provided by a university qualified health coach. GHS health coaches collect socio-demographic characteristics (gender, date of birth, residential postcode, highest level of education obtained, current employment status, language spoken at home and Indigenous status [25]). The postcode of participants is then used to determine Socio-Economic Indexes for Areas (SEIFA), and represent as quintile of relative area disadvantage [26]. The postcode is also used to compute Accessibility-Remoteness Index of Australia Plus (ARIA) [27], a measure of remoteness. All participants were also asked how they heard about GHS (referral source).

### Ethics approval

Informed consent was obtained from all participants prior to their information being included in this study. The study was approved by the University of Sydney Human Research Ethics Committee (Ref. no. 02-2009/11570 and 20110906/14113).

### Statistical analysis

Statistical analyses were conducted using SPSS 19.0 (IBM SPSS Inc. 2011). A media exposure variable was created; low exposure periods included weeks when there was no paid media, medium exposure periods which included some mass-media but did not include television, and high exposure periods which included television advertising. Chi-square tests were performed to examine 1) the relationship between the time of the survey and participant socio-demographic variables and GHS mass-media awareness, knowledge, behaviours and beliefs, and 2) the relationship between referral source and participant socio-demographic variables and participant type. Adjustment of significance method was made using the Bonferroni method [28]. Logistic regression models were computed to examine the association between 1) unprompted vs prompted awareness and actions, and socio-demographic variables, and 2) referral sources, media exposure and service levels (information-only vs coaching).

## Results

### Campaign tracking survey

The profile of survey participants was similar across the three survey periods (data not shown). The majority were female (54.7%); aged over 45 years (67.3%); were employed part or full time (55.2%); were non-Aboriginal (97.9%); spoke English at home (93.4%); had obtained a certificate, diploma, bachelors degree or higher (55.3%); had a household income of greater than AUS$50,000 (58.1%); and were from Sydney (61.0%); further 47.3% of respondents were from the lowest two quintiles of area disadvantage. Approximately 52.6% were classified as being overweight or obese according to their Body Mass Index (BMI); and 49.3% reported that they were ‘more’ or ‘much more’ physically active than others their age. Overall, the profile of survey participants is similar to the target group of GHS and therefore the target audience of the campaign, in terms of their BMI and physical activity levels.

The impact of the GHS campaign on awareness is shown in Table 2. Unprompted awareness increased from 0.0% at baseline to 31.8% at time period 2, and 5.9% at time period 3 (all p < 0.001), and prompted awareness of the GHS increased from 2.5% to 23.7% and 19.8% (all p < 0.001). Understanding of the GHS mass-media communications messages increased from 10.2% at baseline to 31.8% and 32.2% at follow up (all p < 0.001). There was also an increase in survey respondents reporting that they had undertaken action as a result of seeing the advertising (from 2.4% to 4.9% and 3.8%, p < 0.05); more respondents reported that they had called GHS, reviewed the website or encouraged others to call GHS (from 0.0% to 4.0% and 7.7%, not significant); there was a significant increase in knowledge that the service had a free call number (from 22.4% to 35.4% and 38.1%, all p < 0.001) and that the service was provided by a Government agency between baseline and time period 3 (from 87.5% to 95.3%, p < 0.05). There was a moderate but significant increase in the belief that the GHS would be effective or very effective (from 49.4% to 52.9% and 53.0%, p < 0.05) but there was no increase in the belief that the GHS was of personal relevance (data not shown).

**Table 2:** Unprompted and prompted awareness of the GHS campaign by time periods

	**Time period 1 n = 1,544**		**Time period 2 n = 1,500**		**p-value**^**#**^	**Time period 3 n = 1,500**		**p-value**^**Ŧ**^
	**n**	**%**	**n**	**%**		**n**	**%**	
**Unprompted awareness of generic advertising related to healthy lifestyles**								
Yes	938	61.7	1113	75.6	**	1009	69.3	**
No	582	38.3	360	24.4		446	30.7	
**Unprompted awareness of specific advertising related to healthy lifestyles**								
Get Healthy Service (GHS)	0	0.0	154	31.8	**	44	5.9	**
National “Measure up” campaign	204	28.8	118	24.4		261	34.8	
Go for 2 & 5 national fruit/vegetable campaign	15	2.1	51	10.5		12	1.6	
Commercial & other message recall [unrelated]	490	69.2	161	33.3		434	57.8	
**Prompted awareness of GHS on Television**								
Prompted awareness of GHS advertisement	38	2.5	348	23.7	**	291	19.8	**
No awareness	1495	97.5	1122	76.3		1180	80.2	
**Prompted awareness of GHS from Print medium**								
Prompted awareness of GHS advertisement	101	6.7	141	9.7	*	119	8.2	NS
No awareness	1408	93.3	1317	90.3		1328	91.8	
**Prompted awareness of GHS from Magazines**								
Prompted awareness of GHS advertisement	60	3.9	95	6.5	*	87	5.9	*
No awareness	1464	96.1	1366	93.5		1376	94.1	
**Prompted awareness of GHS on Radio**								
Prompted awareness of GHS advertisement	80	5.3	131	8.9	**	130	8.9	**
No awareness	1436	94.7	1341	91.1		1338	91.1	

# Significance value for comparison time period 1 to time period 2 (pre- to post-campaign),chi-square test for categorical data and one-tailed *t*-test for means.

**Ŧ** Significance value for comparison time period 1 to time period 3 (pre- to post-campaign),chi-square test for categorical data and one-tailed *t*-test for means.

** Significant p < 0.001; * Significant p < 0.05, NS not significant.

Multivariate logistic regression examining which participants were more likely to report unprompted and prompted awareness of the GHS campaign (data not shown) found that those who had a post-school education were more likely than those with high school education level to report unprompted campaign awareness (AOR: 1.6, 95% CI: 1.1-2.3, p < 0.05). Those respondents who lived outside of Sydney were significantly less likely to report unprompted campaign recall than those who lived in Sydney (AOR: 0.7, 95% CI: 0.5-1.0, p < 0.05). In relation to prompted campaign awareness, those in the least advantaged quintiles of advantage were significantly less likely to report prompted campaign recall (AOR: 0.9, 95% CI: 0.7-1.0, p < 0.05) and those who were overweight and obese were significantly more likely to report prompted campaign recall (AOR: 1.2, 95% CI: 1.0-1.4, p < 0.05).

### GHS usage data

Between 1 July 2010 and 30 June 2011, a total of 7,100 adults registered with the GHS of which 6,375 (89.8%) participants consented for their information to be included for research and evaluation purposes. The majority were female (73.1%), aged 30 – 59 years (64.1%), employed (58.6%), spoke English at home (91.9%), and lived in major cities (59.9%), 43.4% had a high school education, 2.8% identified as being Aboriginal and 44.5% were from the lowest two quintiles of disadvantage (data not shown). Table 3 details the pattern of mass-media type cited by participants as their primary referral source. Among all types of mass-media, television was the most commonly cited referral source across genders, age groups, education levels, employment status, language groups, SEIFA, region classification, and participant type (all p < 0.001). Press/print advertising was the second most commonly cited mass-media referral source by both males and females, participants aged over 50 years, those who spoke a language other than English at home, Aboriginal participants, information participants, and those in the lowest two quintiles of advantage. Information in the mail was the third most commonly cited mass media referral source, in particular by those aged over 50 years, not employed, in the lowest two quintiles of socioeconomic advantage, those from a location other than a major city and coaching participants. When comparing the proportions of calls to the GHS during campaign and non-campaign periods, it was found that during periods when mass-media advertising was used to promote the GHS, there were approximately 73 information participants and 95 coaching participants recruited per week, 4 times and 2.5 times as many as when there is no mass-media advertising respectively.

**Table 3:** Socio-demographic profile of participants by type of referral source (mass-media channels and other)

		**Mass-media**										**Other**^**#**^		**p-value**
		**TV**		**Radio**		**Press/Print**		**Web**		**Mail out**				
		**n**	**%**	**n**	**%**	**n**	**%**	**n**	**%**	**n**	**%**	**n**	**%**	
**Gender**	Female	2074	45.4	39	0.9	621	13.6	280	6.1	519	11.4	1036	22.7	<0.0001**
	Male	854	51.6	34	2.1	237	14.3	112	6.8	161	9.7	258	15.6	
**Age Groups**	18-49 years	1859	54.8	50	1.5	313	9.2	284	8.4	213	6.3	676	19.9	<0.0001**
	50+ years	1069	37.8	23	0.8	545	19.3	108	3.8	467	16.5	618	21.8	
**Education**	High school education	1267	46.9	32	1.2	457	16.9	104	3.8	332	12.3	510	18.9	<0.0001**
	Other	1654	47.1	41	1.2	399	11.4	288	8.2	346	9.9	782	22.3	
**Employment**	Employed	1728	47.6	62	1.7	458	12.6	285	7.9	297	8.2	798	22.0	<0.0001**
	Other	1194	46.2	11	0.4	396	15.3	106	4.1	382	14.8	494	19.1	
**Language**	English	2746	47.2	71	1.2	780	13.4	354	6.1	643	11.1	1223	21.0	0.001**
	Other	182	44.6	2	1.2	78	19.1	38	9.3	37	9.1	71	17.4	
**Indigenous**	No	2837	46.9	73	1.2	828	13.7	380	6.3	671	11.1	1259	20.8	0.074
	Yes	91	51.4	0	0	30	16.9	12	6.8	9	5.1	35	19.8	
**SEIFA**	1st/2nd/3rd-quintile - most advantaged	1711	48.7	41	1.2	474	13.5	249	7.1	306	8.7	733	20.9	<0.0001**
	4th & 5th-quintile-most disadvantage	660	43.1	20	1.3	219	14.3	81	5.3	204	13.3	348	22.7	
**Region**	Major Cities	1851	49.6	59	1.6	541	14.5	280	7.5	313	8.4	687	18.4	<0.0001**
	Other	1077	43.2	14	0.6	317	12.7	112	4.5	367	13.7	607	24.3	
**Participant type**	Information participant	1211	47.5	33	1.3	410	16.1	221	8.7	231	9.1	442	17.3	<0.0001**
	Coaching participant	1717	46.7	40	1.1	448	12.2	171	4.7	449	12.2	852	23.2	

# Other sources of referral include - friends/family, health professional and general practitioner referral, workplace/employer and other sources.

** Significant at Bonferroni adjusted p-value of 0.003.

The results of a logistic regression analysis (data not shown) examining associations between socio-demographic characteristics, participant type and the likelihood of citing mass-media as the referral source showed that males (AOR: 1.44, 95% CI: 1.27-1.64), Aboriginal callers (AOR: 1.46, 95% CI: 1.02-2.07), and those who contacted the GHS requesting information only (AOR: 1.49, 95% CI: 1.33-1.67) were significantly more likely to cite mass-media advertising as their source of referral. Participants who were significantly less likely to cite mass-media as a referral source were aged 50+ years (AOR: 0.59, 95% CI: 0.53-0.66), had a tertiary education (AOR: 0.84, 95% CI: 0.75-0.95), were from the lowest two quintiles of advantage (AOR: 0.90, 95% CI: 0.79-1.00), or lived in places other than major cities (AOR: 0.63, 95% CI: 0.57-0.70).

The relationships between media exposure, referral sources and registering as coaching participants are shown in the multiple logistic regression Models 1–6 of Table 4. The models are presented in the order in which they were developed. Models 1 and 2 present unadjusted associations between media exposure and referral sources and registering as a coaching participant. Models 3 to 5 present adjusted associations for gender, age, Aboriginal status, language, education level, socio-economic status, and regionality. The final Model 5 includes all the socio-demographic, referral sources and media campaign exposure. The independent and strength of associations between referral sources, media campaign exposure and likelihood of registering as coaching participants remained relatively stable even after adjusting for socio-demographic variables. Odds ratios across all the models clearly show that when there were high levels of media exposure (which included a television component), the recruitment of participants into the coaching program was less likely than when there was no media exposure (AOR: 0.8, 95% CI: 0.6-0.9). The final model, Model 5 further shows that, those participants who cited press, web and television as their referral source were significantly less likely to register as coaching participants when compared to participants who cited other “secondary” referral sources such as health professional referral, family and friends and their workplace (AOR: 0.6, 95% CI: 0.5-0.7, AOR: 0.4, 95% CI: 0.3-0.5, AOR: 0.7, 95% CI: 0.7-0.9, respectively). There was no difference between mail out information cited as a referral source and other “secondary” referral sources and coaching participant recruitment.

**Table 4:** Adjusted Odds Ratio (AOR) and 95% confidence intervals (CI) for likelihood of registering as coaching participants

		**Total N**	**Coaching**		**Model 1 Media exposure**		**Model 2 Referral sources**		**Model 3 SE, referral sources**		**Model 4 SE, media exposure**		**Model 5 SE, media exposure & referral sources**
			**n**	**%**									
**Gender**	Female (ref)	4661	2846	61.1									
	Male	1714	873	50.9					0.72 (0.64-0.81)	**	0.70 (0.63-0.79)	**	0.72 (0.64-0.81)
**Age Groups**	18-49 years (ref)	3485	2021	58.0									
	50+ years	2890	1698	58.8					0.97 (0.87-1.09)	NS	1.00 (0.89-1.11)	NS	0.98 (0.87-1.09)
**Aboriginal**	Non Aboriginal (ref)	6197	3609	58.2									
	Aboriginal	178	110	61.8					1.23 (0.90-1.68)	NS	1.13 (0.83-1.55)	NS	1.21 (0.88-1.66)
**Language**	English (ref)	5858	3473	59.3									
	Other	517	246	47.6					0.96 (0.78-1.19)	NS	0.94 (0.76-1.16)	NS	0.97 (0.78-1.20)
**Education**	High school education (ref)	2714	1551	57.1									
	Tertiary	3541	2155	60.9					1.24 (1.12-1.39)	**	1.25 (1.12-1.39)	**	1.24 (1.12-1.39)
**Employment**	Employed (ref)	3664	2096	57.2									
	Other	2588	1609	62.2					1.28 (1.14-1.43)	**	1.27 (1.14-1.42)	**	1.27 (1.13-1.42)
**SEIFA**	1st/2nd/3rd-quintile - most advantaged (ref)	3552	2122	59.7									
	4th & 5th-quintile-most disadvantage	2823	1597	56.6					0.93 (0.83-1.03)	NS	0.94 (0.84-1.04)	NS	0.93 (0.83-1.03)
**Region**	Major Cities (ref)	3770	2234	59.3									
	Other	2605	1485	57.0					0.93 (0.83-1.04)	NS	0.97 (0.87-1.08)	NS	0.93 (0.83-1.04)
**Referral**	Other (ref)	1294	852	65.8									
	Radio	73	40	54.8			0.63 (0.39-1.01)	NS	0.72 (0.44-1.16)	NS			0.74 (0.45-1.19)
	Press	858	448	52.2			0.57 (0.48-0.68)	**	0.60 (0.49-0.71)	**			0.60 (0.50-0.72)
	Web	392	171	43.6			0.40 (0.32-0.51)	**	0.40 (0.32-0.51)	**			0.41 (0.32-0.52)
	Mail out	680	449	66.0			1.02(0.83-1.23)	NS	1.03 (0.84-1.25)	NS			1.00 (0.82-1.22)
	TV	2928	1717	58.6			0.74 (0.64-0.84)	**	0.76 (0.66--0.87)	**			0.70 (0.69--0.91)
**Media exposure**	None(ref)	677	454	67.1									
	Medium level	1599	966	60.4	0.75 (0.62-0.91)	*					0.78 (0.64-0.94)	*	0.88 (0.72-1.06)
	High level	4099	2299	56.1	0.63 (0.53-0.75)	**					0.69 (0.58-0.82)	**	0.76 (0.63-0.91)

Ref: Reference. SEIFA: Socio-Economic Index for Areas, SE Socio-economic variables ** Significant at p < 0.001, * Significant at p < 0.05, NS: not significant.

## Discussion

The GHS mass-media marketing campaign was effective at raising awareness about GHS, at increasing GHS knowledge particularly in relation to the GHS being provided by a Government agency and that it could be contacted through a freecall phone number. Results from the current study are consistent with previous evidence that mass-media can have a positive impact on service utilisation [10], and demonstrate an increase call volume to GHS, particularly by males, those aged 30 – 39 year old, those who were employed, those from the lowest two quintiles of advantage, those living in major cities and those registering as information participants. This study also demonstrated that during periods of GHS mass-media advertising four times and 2.5 times as many information and coaching participants respectively were recruited compared to when GHS relied on other non-media promotional and referral mechanisms.

The increase in the levels of awareness (prompted and unprompted) associated with the GHS following the periods of mass-media advertising are in line with other public health related mass-media campaigns [29-31]. Interestingly there was higher unprompted awareness at time period 2 (31.8%) compared to time period 3 (5.9%) which could be accounted for by national healthy weight campaign airing prior to time period 2, whilst only aired in NSW for four weeks, it had a high level of media exposure. Those participants who lived in locations other than Sydney were more likely to report prompted awareness of the GHS advertising, whereas those from the lowest two quintiles of socio-economic advantage were less likely to report prompted awareness. This indicates the success of the mass-media advertising at promoting GHS to those outside of Sydney but not being as successful at reaching those in the lowest quintiles of socio-economic advantage; confirming the important role that mass-media advertising has in facilitating rural accessibility [19].

It is well reported that raising awareness may be a mediator of taking action and that those who are more aware are also more amenable to engaging in behaviour change or health-care seeking behaviours [32-36]. Respondents to the tracking survey reported having undertaken some behavioural lifestyle changes as a result of seeing the GHS mass-media advertising, including choosing to be more active and eating more healthy foods; the magnitude of change was small (2.5%), but still significant. This is good news for both promoting healthy lifestyle behaviours and GHS service utilisation. With the significant increases in awareness brought about by the implementation of the GHS advertising campaign, the results clearly show that GHS mass-media advertising has a substantial impact on the call volume to the GHS as well as influencing positive changes towards increasing physical activity and choosing to eat healthier foods, effects similar to the “tip of the iceberg” of quitting activity experienced as a greater proportion of calls to the Quitline [37].

The inclusion of GHS service utilisation data in this study recognises the importance of campaign related process evaluation, including collecting information pertaining to a call to action by any social marketing campaign [32]. Moreover, the mass-media campaign was particularly effective at reaching males, 18–49 year olds and those in the lowest two quintiles of advantage. However, mass-media was also cited as the main referral source for those who opted for information-only package, whereas those who registered in the coaching program were more likely to have identified another source of referral such as family or friends, workplaces, general practitioner and health professional referrals. This is similar to the experience of telephone-based smoking cessation Quitlines where greater recruitment to counselling occurs when the advertising investment was lower [37] and other referral mechanisms are relied upon.

The findings of this study also provide important evidence in relation to the effectiveness of specific components of the mass-media campaign. The information from service usage data lends support to the general effectiveness of the television advertising across all socio-demographic segments at recruiting GHS participants in general. Second to television advertising, press advertising was also particularly effective at recruiting those aged 50 + years, those in the 4th and 5th quintile and those who identified as being from Aboriginal communities and those who speak a language other than English at home. This provides valuable support to the need to ensure the integration of campaign messages aimed at vulnerable communities using culturally specific mechanisms. In early 2010 GHS press advertisements were adapted to be more appropriate to Aboriginal communities and culturally and linguistically diverse communities and placed in Aboriginal and other community specific publications. The results from service usage data support the effectiveness of these initiatives at promoting participant engagement from these communities. The distribution of GHS information in specifically targeted households was also particularly effective at recruiting participants over the age of 50 years, those who were not employed, in the lowest two quintiles of advantage and those who lived outside of major cities, reflecting the targeted placement of this promotion.

The question of which types of mass-media are most effective at recruiting coaching participants is also of importance; as it is the evidence-based coaching program that is more likely to change the long term chronic disease risk factor profile of adults, compared to those registered as information-only participants. In this regard, when compared to other types of mass-media referral sources, mail out was most effective at recruiting individuals to the coaching program (in the presence of mass-media exposure). This study also notes that it is sources other than mass-media that are most effective at recruiting coaching participants. That is, recommendations from family and friends, and even the employer or workplace, as well as direct referrals by general practitioners and other health professionals are critical in “introducing” a potential participant to the GHS. These referral mechanisms, like the information provided in the letter box, provide an opportunity to explain the GHS in further detail and result in a greater likelihood of a participant registering for the coaching program.

The current findings provide opportunities for improving the ways in which mass-media and GHS itself can work to increase GHS coaching participation rates. In particular, the authors draw on the experience of Quitline promotion as a tobacco cessation support service with varying service levels, which are undertaken within a comprehensive approach to tobacco control mass-media that include both “push” and “pull” style campaigns. The “push” style campaigns tend to promote the health consequences of smoking and are supported by “pull” campaigns that offer advice/counselling or suggest a call to action [37,38]. Within this context, a study has shown that the “pull” style advertising is successful at recruiting participants to the ongoing support offered by the Quitline, rather than just opting for the information component [37]. The GHS as a support service is primarily being promoted in isolation of any public health education about the health consequences of physical inactivity, unhealthy eating and overweight/obesity. Thus, recruitment of coaching participants through mass-media may be more successful when positioned within a wider campaign. The promotion of the GHS within the national healthy weight campaign for four weeks during the study period did not allow for adequate exploration of this hypothesis. Further, the success of the letterbox and magazine pamphlet insert was as effective as other secondary referral sources at recruiting coaching participants. This, alongside the broader mass-media campaign advertising is a low cost promotion that can be targeted to particular populations and warrants repeat use. Specifically-developed print advertising was successful at recruiting Aboriginal and culturally and linguistically diverse communities to GHS and if further refined to include more detail about the availability of coaching support may be successful at increasing the number of registrants to coaching program.

The results of this study also have practical implications for the design of the GHS and the opportunity to facilitate greater recruitment into coaching among those who contact GHS. The current introductory script to all callers promotes the information kit as the first option, followed by the health coaching program. The coaching program could be promoted as the default service option and for those that are reluctant to join, the information kit could be provided with the addition of a follow up phone call persuading coaching program involvement. Currently 5% of information participants re-contact the GHS and opt to join the coaching program, opportunities to maximise this conversion rate would be beneficial.

### Limitations

The evaluation of the GHS mass-media campaign has certain limitations, namely there may be possible sample bias due to the tracking survey only being offered to those who have landlines; and the design was uncontrolled, which is a limitation common to many population based campaign evaluations [32]. The inclusion of GHS service usage data, however, provides an opportunity to corroborate and support the information obtained in the tracking survey.

## Conclusions

Mass-media campaigns, led by television advertising, are effective at increasing awareness, knowledge and beliefs regarding the GHS. There is also a clear relationship between mass-media and the recruitment of GHS participants. Whilst television with its universal reach and visibility, is required to lead GHS recruitment efforts, it is of public health importance that auxiliary mass-media, including tailored press advertising and targeted letterbox information is used to recruit particularly vulnerable communities; further opportunities to have the service “introduced” to the participant by second-party referral sources are important to ensure coaching participation. The utility of the GHS coaching component could be further promoted with changes to the GHS introductory script and through general mass-media campaigns promoting healthy weight which incorporate the GHS in a supportive “pull” support style campaign.

The results of this study have implications for broader service utilisation; mass media strategies are effective at ensuring universal reach and when supplemented by other referral channels such as health professionals and targeted promotional activities, the mix of strategies can maximise recruitment and engagement in population-wide lifestyle change programs.

## Competing interests

The authors acknowledge that there are no competing interests.

## Authors' contributions

BOH was responsible for the design, drafting, analysis of data, and drafting and editing of the manuscript. AB was responsible for the reviewing and editing the manuscript. PP was responsible for reviewing and editing the manuscript and overseeing the data analysis. All authors read and approved the final manuscript.

## Pre-publication history

The pre-publication history for this paper can be accessed here:

http://www.biomedcentral.com/1471-2458/12/762/prepub
